# Quercetin for myocardial ischemia reperfusion injury: A protocol for systematic review and meta-analysis

**DOI:** 10.1097/MD.0000000000020856

**Published:** 2020-06-26

**Authors:** Liying Lu, Xiaocong Ma, Jinghui Zheng, Lijuan Li, Wenna Yang, Yixuan Kong, Jie Wang

**Affiliations:** a Ruikang Affiliated Hospital of Guangxi University of Chinese Medicine; b Department of Geriatrics, Ruikang Affiliated Hospital of Guangxi University of Chinese Medicine, Nanning, Guangxi, China.

**Keywords:** meta-analysis, myocardial ischemia reperfusion injury, quercetin, systematic review

## Abstract

**Background:**

At present, there is no effective therapy for preventing myocardial ischemia reperfusion injury (MIRI), and it is inevitable. The methods how to effectively decrease MIRI have attracted the attention of medical researches in recent years. Quercetin is a part of natural flavonoids in plant polyphenols. Many studies have found that quercetin has a positive effect on MIRI.

**Methods:**

In order to clarify the effectiveness and potential mechanisms of quercetin for MIRI animals, we searched for animal studies of quercetin for MIRI in Wanfang data Information, Chinese National Knowledge Infrastructure, VIP information database, China Biology Medicine disc, EMBASE, PubMed, and Web of Science. Participant intervention comparator outcomes of this study are as flowing: P, rats in MIRI; I, received quercetin treatment merely; C, received only vehicle or no treatment; O, Main outcomes are myocardial infarction size and markers of myocardial injury. Additional outcomes are serum indices or protein levels tied to the mechanisms of quercetin in myocardial l/R injury. Review Manager 5.2 software and Stata14.0 will be used for data analysis. SYRCLE's risk of bias tool will be used for risk of bias analysis of animal studies.

**Discussion:**

This preclinical systematic review and meta-analysis will evaluate the effects and mechanisms of quercetin for MIRI animals, and provide more evidence-based guidance for transforming basic research into clinical treatment.

**Trial registration:**

INPLASY202050067, registered on 16/5/2020.

## Abstract 2

Supplemental Digital Content is available in the text

## 1 Introduction

Cardiovascular disease has brought a great health and economic burden for society, and it has been 1 of the 10 main causes of death in the world.^[1]^ Ischemic heart disease (ie, coronary heart disease) is a common clinical disease caused by insufficient myocardial blood and oxygen. Restoring myocardial blood supply is the fundamental measure for treatment, and reperfusion therapy is the most effective method for clinical treatment of ischemic heart disease. Thrombolytic agent, percutaneous coronary intervention and coronary artery bypass grafting are used for reperfusion in clinical practice.^[2]^ Although revascularization as early as possible is the most effective way to reduce myocardial ischemic injury, it is accompanied with myocardial ischemia reperfusion injury (MIRI). MIRI is caused by calcium overload, apoptosis, mitochondrial damage, increased generation of oxygen free radicals and other biological processes.^[3]^ Though the myocardial reperfusion has been improved with more timely and effective reperfusion, and more advanced percutaneous coronary intervention techniques, antiplatelet, and antithrombotic agents used to maintain the patency of infarct-related coronary arteries, there is still no effective therapy to prevent MIRI.^[4]^ How to effectively prevent and reduce MIRI is the focus of medical research in recent years.

Flavonoidqs are widely found in fruits, vegetables, wines, and Chinese herbal medicine. Flavonoids play an important role on antioxidative, anti-inflammatory, and antitumor.^[5,6]^ Quercetin is a part of natural polyphenol flavonoids in plants^[7]^ and it is rich in Camellia sinensis.^[8]^ Considerable quantities of quercetin were found in kale and red onions contain.^[9]^ Quercetin exerts multiple pharmacological effects, such as antidiabetic, antioxidative, anti-inflammatory, and antitumor.^[10–12]^ Epidemiological studies have shown that increasing the intake of quercetin properly can reduce the risks of certain chronic diseases such as osteoporosis, cardiovascular disease and diabetes.^[13–16]^ Many animal experiments have shown that quercetin has a positive effect on MIRI. In order to clarify the effectiveness and potential mechanism of quercetin for MIRI animals, we will conduct a preclinical systematic review, which is of great significance for transforming basic research into clinical treatment.

## 2 Methods

### 2.1 Ethics and dissemination

This preclinical systematic review and meta-analysis is a secondary research based on previously published studies. Therefore, ethical approval and informed consent are not required for this study. This preclinical systematic review and meta-analysis will be published in a peer-reviewed journal.

### 2.2 Eligibility criteria

**Participant or population:** rats in MIRI.

**Intervention:** received quercetin treatment merely

**Comparator:** only received vehicle or no treatment.

Study designs to be included: randomized controlled studies.

Type of outcomes: main outcomes are myocardial infarction size and markers of myocardial injury. Additional outcomes are serum indices or protein levels relative to the mechanisms of quercetin for MIRI.

Data Extraction: Studies will be included for this systematic review when they suit the flowing terms:

(1) animal studies that evaluated the administration of quercetin for MIRI (cell studies, case reports, reviews, only abstracts, and comments will be excluded);
(2) Only the treatment for analysis of quercetin intervention will be received; control group intervention received only vehicle or no treatment;
(3) Main outcomes were myocardial infarction size and myocardial injury marker, addition outcomes were serum indices or protein levels tied with the mechanisms of quercetin in MIRI.

Exclusion criteria: studies without full text, cell studies, case reports, reviews, abstracts, and comments will be excluded. The studies administered other additional pharmacological treatment, without the predetermined outcome index and without MIRI animal models will not be eligible for inclusion in this study. Full-text articles will be excluded when they meet:

(1) no full text;
(2) no quercetin intervention;
(3) no control group;
(4) no MIRI models;
(5) combination with other treatment;
(6) no available data.

**Figure 1:**
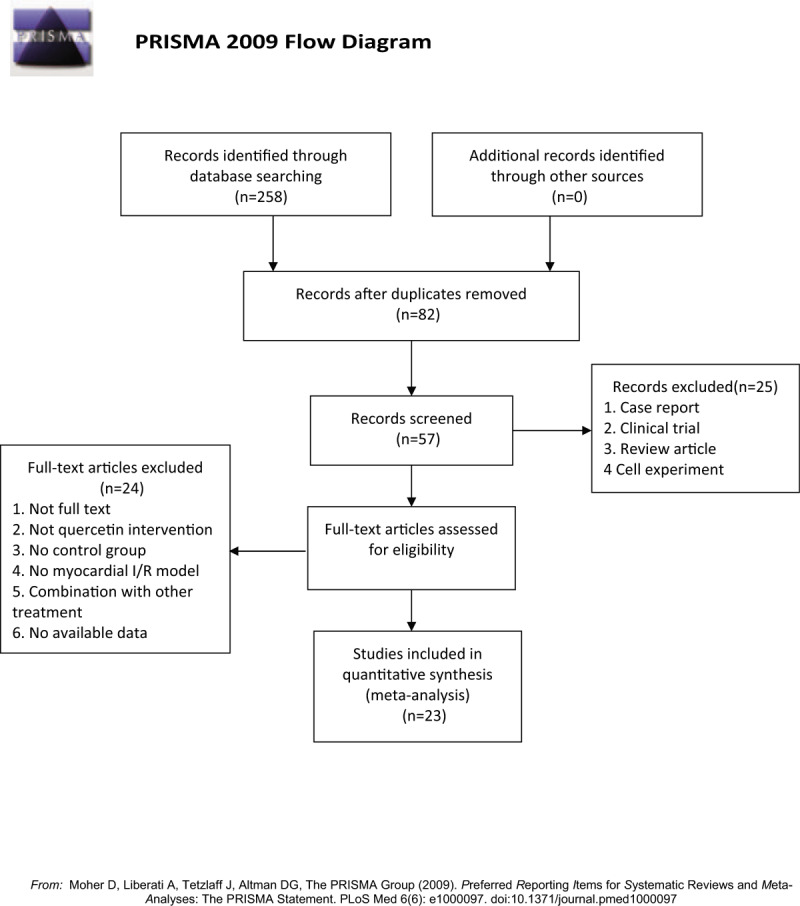
Screening process.

The study selection is shown as Figure 1.

### 2.3 Information sources

We searched for animal studies of quercetin for MIRI in Chinese National Knowledge Infrastructure, EMBASE, PubMed, VIP information database, Web of Science, China Biology Medicine database, and Wanfang data Information. We set the search range from inception to March 2020. We also obtain the suitable studies from grey literature compiles studies, as well as the sites of animal research organizations, Google Scholar, and Baidu Scholar, which are not covered in the databases mentioned. The retrieval keywords as followings: hupisu, quercetin, myocardial ischemia reperfusion, and myocardial infarction. Search strategy to be used of PubMed for this study can be found in Supplementary Materials1.

### 2.4 Data collection and analysis

Two independent reviewers will conducted selection process. If there is disagreement, we will have a group discussion. The following information will be extracted from the literature:

(1) year of publication and name of first author;
(2) species, gender, number and weight for animals;
(3) methods for myocardial I/R model, including ischemic time, perfusion time, and coronary artery occluded;
(4) methods for anesthesia induction;
(5) intervention time, dosage, and route of administration for treatment and control group;
(6) the main outcome measures, addition outcome measures and differences of intergroup.

If different doses of drugs were used in the study, the results of the highest dose would be extracted; when outcomes measured at different time points, only the data of the measured test would be recorded; we will contact the author to obtain raw data, if data were only show as graphs. Measure numerical will be valued by digital ruler software when there are no response from authors.

### 2.5 Data synthesis

Review Manager 5.2 software and Stata14.0 will be used for data synthesis. Standardized mean difference will be used for estimate of the combined effect sizes .The heterogeneity assumption will be evaluated by *I*^2^ statistics, (*I*^2^ > 50%, means large heterogeneity; 25% < *I*^2^ ≤ 50%, means medium heterogeneity; and 0 ≤ *I*^2^ ≤ 25%, means small heterogeneity). If there is no evidence showed large heterogeneity, a fixed effect model analysis will be used; otherwise, random effect model analysis will be chosen after excluding the sources of heterogeneity. 95% confidence intervals of all results will be calculated, the significance will be determined by *Z*-test, with a *P* value < 0.05 as significant level. Metaninf of Stata14.0 will used for assessing risk of bias of individual studies.

### 2.6 Quality assessment/risk of bias analysis

The study quality assessment will be independently valued by 2 authors with the Collaborative Approach to Meta-Analysis and Review of Animal Data from Experimental Studies. Two authors will independently evaluate the quality of study using the 10-point scoring scale: A, peer reviewed publication; B, control of temperature; C, random allocation to treatment or control; D, blinded induction of model; E, blinded assessment of outcome; F, use of anesthetic without significant intrinsic cardioprotective activity; G, animal model (aged, diabetic, or hypertensive); H, sample size calculation; I, compliance with animal welfare regulations; J, statement of potential conflict of interests. Risk of bias analysis: SYRCLE's risk of bias tool will be used for animal studies.

### 2.7 Subgroup analysis and sensibility analysis

Subgroup analyses, which are exists for animals’ species, age, gender, intervention time, dosage and route of administration, ischemic time and perfusion time, is a method will be used to find the possible sources of heterogeneity. In order to screen out the influencing factors that lead to heterogeneity, meta-regression will be used to reflect the relationship between 1 or more explanatory variables and the outcome variable.

### 2.8 Reporting bias

Reporting bias may affect the results of systematic reviews. The control of publication bias is more difficult and has a greater degree of impact. Therefore, the identification and processing of publication bias is an important step in systematic reviews. The funnel plot, a kind of visualization method, will be used to identify reporting bias.

## 3 Discussion

This meta-analysis will evaluate the effects and mechanisms of quercetin for MIRI animals, and provide more evidence-based guidance for transforming basic research into clinical treatment. In order to make more suitable studies concluded, we have searched several well-known international databases and commonly used databases in China. Wong's findings^[17]^ highlighted the importance of including trial registries in the grey literature search. In our study, not only the commonly used databases at home and abroad are searched, but also the gray literature. However, the studies we include only Chinese and English studies, which still have limitations. What is more, we have developed suitable plans to deal with the risk bias, reporting bias and heterogeneity that may occur in this study.

Preclinical researches have provided key resources for clinical development, but most medical interventions introduced into clinical development have proved to be unsafe or ineffective. The prominent explanation of leading to such a result is that there are some defects in preclinical searches.^[18]^ Preclinical systematic review and meta-analysis of quercetin for MIRI can not only help to further understand the therapeutic effects and mechanisms of quercetin on disease, but also helps to lay the foundation for future researches and improve the success rate of the transformation of basic research into clinical medicine.

## Acknowledgments

Thanks are due to Zhuomiao Ye for assistance with this study and valuable discussion.

## Author contributions

Liying Lu designed this research and drafted the manuscript, Xiaocong Ma tested the feasibility of the study, Lijuan Li contributed to the development of the selection criteria, and the risk of bias assessment strategy, and Jinghui Zheng read, provided feedback and approved the final manuscript. All authors approved the final version of the manuscript.

## Supplementary Material

Supplemental Digital Content
