# Supplementary material for: Quercetin for myocardial ischemia reperfusion injury: A protocol for systematic review and meta-analysis
Source: Medicine (Baltimore). 2020 Jun 26;99(26):e20856. doi: 10.1097/MD.0000000000020856 (PMC7328958; doi:10.1097/MD.0000000000020856)
Supplement: Supplemental Digital Content [file medi-99-e20856-s001.doc]

**(((Quercetin"[Mesh]) AND myocardial ischemia reperfusion) OR myocardial I/R injury) OR myocardial infarction**
